# P02.39. Leveraging grant awards to enhance the research infrastructure at a CAM institution

**DOI:** 10.1186/1472-6882-12-S1-P95

**Published:** 2012-06-12

**Authors:** K Pohlman, L Carber, R Vining, T Devlin, R Rice, S Salsbury, L Corber, M Hondras, C Long, C Goertz

**Affiliations:** 1 Palmer College of Chiropractic, Davenport, USA

## Purpose

Successful scientific investigations at CAM institutions are often hampered by limited research infrastructure. Enhancing the clinical trial capabilities of our chiropractic research center was one goal of 2 NIH/NCCAM developmental center grants.

## Methods

Web applications were enhanced and customized based on individual project needs and trial team requests. Templates were built to support quick development and standard functionality of project web applications. We also developed standardized processes to effectively provide project-specific participant management, data collection, staff training, and quality control.

## Results

Web applications included: the Centralized Participant Database System and Project/Users Permissions System to securely capture participant contact information and control personnel access to web modules; a real-time participant tracking report to monitor recruitment and participant flow; a role-specific Reminder System to track outstanding activities requiring personnel follow-up; and double key-entry verification on web data used for an adaptive treatment allocation algorithm. Templates of these tools can be incorporated into any new trial’s web application. Study protocol templates facilitate timely, accurate translation of research proposals into detailed protocols for IRB applications, DSMC review and training. A revised training plan included role-specific training logs for tracking training objective completion and a thorough certification process for key protocols to ensure accurate and consistent performance. Quality control templates support the informed consent process and fidelity of trial interventions and outcomes assessment. We implemented these enhanced resources in 5 clinical trials. To date, our team has completed 3378 phone screens, conducted 1439 first and 473 second in-person screening visits, enrolled 394 participants, and followed 315 through trial end. The study protocol templates have been used in two additional clinical trials currently undergoing IRB review.

## Conclusion

We expanded our capacity to support multiple, diverse trials with complex methodologies. This is a successful example of a CAM institution successfully leveraging research grant funding to enhance research infrastructure.

